# Blood–Brain Barrier-Targeting Nanoparticles: Biomaterial Properties and Biomedical Applications in Translational Neuroscience

**DOI:** 10.3390/ph17050612

**Published:** 2024-05-10

**Authors:** Evridiki Asimakidou, Justin Kok Soon Tan, Jialiu Zeng, Chih Hung Lo

**Affiliations:** 1 Department of Clinical Neurosciences, University of Cambridge, Cambridge CB2 0QQ, UK; euriasim@gmail.com; 2 Department of Biomedical Engineering, College of Design and Engineering, National University of Singapore, Singapore 117575, Singapore; j.tan@nus.edu.sg; 3 The N.1 Institute for Health, National University of Singapore, Singapore 117456, Singapore; 4 Lee Kong Chian School of Medicine, Nanyang Technological University, Singapore 308232, Singapore

**Keywords:** blood–brain barrier (BBB), BBB permeability, nanoparticle, biomaterial, physicochemical, conjugation, BBB models, microfluidics, BBB disruption, neurodegenerative diseases

## Abstract

Overcoming the blood–brain barrier (BBB) remains a significant hurdle in effective drug delivery to the brain. While the BBB serves as a crucial protective barrier, it poses challenges in delivering therapeutic agents to their intended targets within the brain parenchyma. To enhance drug delivery for the treatment of neurological diseases, several delivery technologies to circumvent the BBB have been developed in the last few years. Among them, nanoparticles (NPs) are one of the most versatile and promising tools. Here, we summarize the characteristics of NPs that facilitate BBB penetration, including their size, shape, chemical composition, surface charge, and importantly, their conjugation with various biological or synthetic molecules such as glucose, transferrin, insulin, polyethylene glycol, peptides, and aptamers. Additionally, we discuss the coating of NPs with surfactants. A comprehensive overview of the common in vitro and in vivo models of the BBB for NP penetration studies is also provided. The discussion extends to discussing BBB impairment under pathological conditions and leveraging BBB alterations under pathological conditions to enhance drug delivery. Emphasizing the need for future studies to uncover the inherent therapeutic properties of NPs, the review advocates for their role beyond delivery systems and calls for efforts translating NPs to the clinic as therapeutics. Overall, NPs stand out as a highly promising therapeutic strategy for precise BBB targeting and drug delivery in neurological disorders.

## 1. Introduction

Neurological disorders such as Alzheimer’s disease (AD), Parkinson’s disease (PD), multiple sclerosis (MS), stroke, and brain tumors manifest with a multitude of clinical symptoms that are distinct for each clinical condition [1,2,3,4]. The most typical symptoms include memory decline in AD, bradykinesia in PD, gait impairment and sensory deficits in MS, speech impairment in stroke, and progressive sensorimotor deficits and seizures in the case of brain tumors [1,5]. Therapeutic intervention against these neurological diseases often requires brain-targeting drug delivery [1,6]. However, drug discovery efforts are often hampered by the large percentage of drugs that are unable to cross the blood–brain barrier (BBB) to target the brain due to their large sizes (>400 Da) and low lipophilicity [7,8]. Hence, circumventing the BBB poses a major challenge and holds paramount importance in the context of treating various neurological disorders.

**Figure 1:**
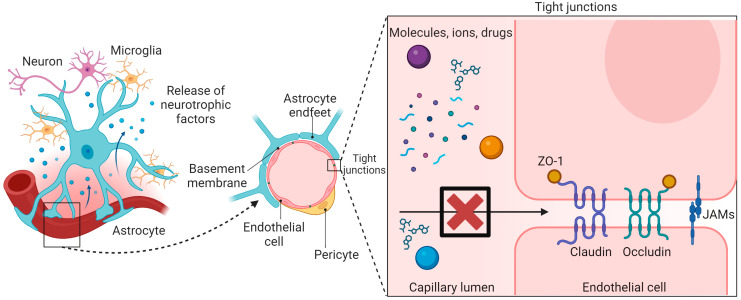
Structure and function of the BBB. The BBB is structurally composed of endothelial cells, the vascular basement membrane, pericytes, and astrocyte end-feet. The endothelial cells of the BBB have tight junctions that lack fenestration and pinocytic activity, thereby preventing most molecules, ions, and drugs from paracellular transport. Tight junctions consist of different integral membrane proteins, namely, claudin, occludin, and junction adhesion molecules (JAMS), and several cytoplasmic accessory proteins including Zonula occludens-1 (ZO-1) and others. This schematic was created with BioRender.com.

The BBB is structurally composed of endothelial cells, the vascular basement membrane, pericytes, and astrocyte end-feet [9] (Figure 1). The endothelial cells of the BBB have tight junctions lacking fenestration and pinocytic activity, thereby preventing most molecules and ions from paracellular transport [10]. Pericytes are vascular mural cells located on the abluminal aspect of endothelial cells and wrapped by astrocyte end-feet, which regulate cerebral blood flow by controlling the capillary vascular tone. Pericytes are important for BBB formation and for downregulation of transcytosis activity when endothelial cells mature [11]. Astrocytes play a crucial role in maintaining BBB integrity by releasing neurotrophic factors and regulating permeability through the secretion of vasoconstrictor or vasodilator mediators [12,13]. Therefore, pericytes and astrocytes affect transportation across the BBB. Drugs primarily penetrate the BBB and access the brain parenchyma through paracellular transport between endothelial cells and astrocytic end-feet or via transcellular transport [7,14]. In transcellular transport, passive and active transport mechanisms are involved. Passive transport relies on the physicochemical properties of the therapeutic agent, such as small size and lipophilicity, facilitating passive diffusion [7,14]. Conversely, active mechanisms occur through carrier- or receptor-mediated processes, requiring energy expenditure [7,14].

Various strategies have been developed to improve drug delivery to the brain by bypassing the BBB [15]. These strategies can be classified into invasive techniques including intracerebral grafts, intrathecal brain delivery, and direct brain injection, and non-invasive techniques such as focused ultrasound, intranasal delivery, and nanoparticle (NP)-mediated delivery [15]. In non-invasive delivery techniques, intranasal delivery is an effective route for administration of small lipophilic molecules to the brain [16], whereby the drugs enters the nasal blood vessels and reach the BBB. NP-mediated delivery, due to NPs’ non-invasive nature, adaptable administration routes, and customizable properties such as stability and capacity to encapsulate both hydrophilic and hydrophobic substances, has emerged as a versatile and promising approach for targeted brain delivery. The size of NPs ranges between one and several hundred nanometers (nm), with optimal sizes of about 10–100 nm for BBB targeting [17]. The primary categories of NPs encompass organic NPs, which are composed of proteins, carbohydrates, lipids, polymers, or other organic compounds; carbon-based NPs, including carbon dots; and inorganic NPs, which comprise metallic, ceramic, and semiconductor NPs [18]. NPs are typically used as delivery vehicles to transport drugs to their target site, either encapsulating the drugs inside the NPs or conjugating them at the surface of the NPs [19,20,21]. Recent advances in nanoformulation systems to further enhance drug delivery and efficacy include soft nanomaterials (e.g., nanogels, and dendrimers), condensed nanomaterials (e.g., noble metals), and natural biodegradable polymers (e.g., chitosan, alginate, starch) [22,23,24]. The versatility of material properties enables the fine-tuning of drug lipophilicity and drugs’ uptake and targeting profiles. This capability enhances drug delivery effectiveness across various contexts [17,25]. Importantly, there are also examples of NPs without any drug conjugation that can serve as therapeutic agents themselves due to the medicinal chemistry of the materials used in their synthesis [19,20,26,27].

In a clinical setting, NPs have been FDA approved for the treatment of various medical conditions such as ovarian, breast, prostate, and lung cancer, leukaemia, Crohn’s disease, rheumatoid arthritis, psoriatic arthritis, and ankylosing spondylitis [25,28]. However, in terms of brain targeting, NPs have only been approved for treatment of MS [25], although they have been used for brain targeting in pre-clinical models and ongoing clinical trials [29]. Hence, there is a need to better understand how to optimize NPs for improved BBB penetration and brain targeting. In this review, we summarize optimization strategies to enhance the BBB penetration efficacy of NPs based on their physiochemical properties and conjugations with functional ligands under different physiological and pathological conditions. We further discuss different in vitro and in vivo BBB models and technologies that can be used to determine the NPs’ ability to penetrate the BBB. Finally, we provide future perspectives on NPs and drug-conjugated NPs as frontiers in nanomedicine and potential personalized medicine, as well as their clinical applications in the treatment of neurological diseases.

## 2. Factors for Optimization of Nanoparticles to Enhance BBB Penetration

**Figure 2:**
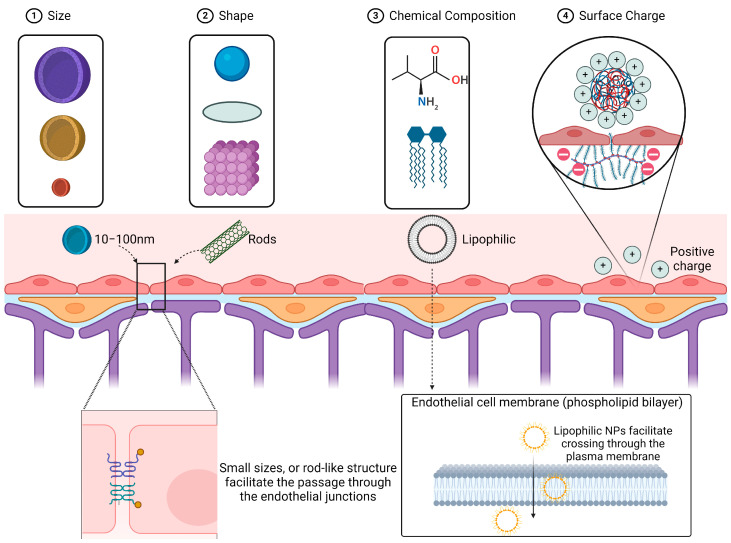
Factors enabling NPs’ BBB penetration. Factors influencing NPs’ ability to penetrate the BBB. The size, shape, chemical composition, and surface charge are four major factors that affect NPs’ BBB penetration capability. Small NPs with a rod-like structure, lipophilic NPs, and NPs with positive charges have a higher capability of crossing BBB endothelial junctions. Schematics were created with BioRender.com.

The physiochemical characteristics of NPs play crucial roles in determining their pharmacokinetic properties and the range of their biomedical applications. These features are unique to each type of NP and are fundamental for interactions with their respective targets in different brain regions. The physicochemical profiles can influence peripheral metabolism and determine the amount of the drugs that can enter the brain. Key factors include the size and shape, chemical composition (lipophilicity, biodegradability), surface charge, and surface modification of NPs (Figure 2).

### 2.1. Size and Shape

One of the key factors that determines the ability of NPs to penetrate the BBB is their size. BBB endothelial cells have tight junctions that allow molecules of less than 400 Da to cross via passive diffusion [7]. Hence, the smaller the size of a NP, the higher its probability of crossing the BBB. Nevertheless, it is important to acknowledge that NPs smaller than 5 nm are prone to elimination by the kidneys. Thus, there exists an optimal size range for NPs, typically between 10 and 100 nm, that facilitates BBB penetration without encountering renal elimination [17]. In one study, the size-dependent biodistribution of gold NPs of four different sizes—10, 50, 100, and 250 nm—administered in healthy male Wistar-derived rats was measured to assess their ability to penetrate the BBB. It was reported that while most of the NPs showed accumulation in the liver and spleen, NPs with a size of 10 nm were the only ones that could be detected in the brain [30]. In another instance, poly(lactic-co-glycolic acid) (PLGA) NPs of different sizes (100 nm, 200 nm, and 800 nm) were administered to a BALB/c nude mouse with TBI; NPs with a size of 100 nm penetrated the greatest depth through the TBI region located in the central region of the parietal lobe [31]. Similarly, the dynamics of methotrexate-loaded polybutylcyanoacrylate NPs with different sizes of 70, 170, 220, and 345 nm across the BBB were investigated via intravenous injection into healthy Sprague–Dawley rats, and NPs less than 100 nm showed penetration through the BBB [32]. While the size of NPs is an important factor for BBB penetration, there is currently no consensus on the optimal size across broad categories of NPs. The shape of NPs also determines their potential to cross the BBB. In most cases, NPs have a spherical morphology, but other shapes like discs, rods, and cubes can also be constructed [33]. The shape of the NPs not only influences their ability to interact with the brain endothelium but also their clearance rate from the brain through blood circulation. Compared to spherical polystyrene NPs, rod-shaped NPs adhere to a greater extent to the brain endothelium and are subsequently transferred into the brain parenchyma, leading to a higher accumulation in the brain [34,35]. Beyond this central interaction, a non-spherical geometry is associated with a lower probability of uptake from peripheral phagocytes, resulting in a higher amount of NPs reaching the central nervous system (CNS) [36].

### 2.2. Chemical Composition (Lipophilicity, Biodegradability)

The chemical composition of nanoparticles, particularly their lipophilicity, significantly influences their ability to penetrate the BBB [37,38]. As mentioned in the previous sections, the BBB consists of endothelial cells which are highly lipophilic. Hence, NPs which are lipophilic in nature can efficiently cross the endothelial monolayer and enable sufficient delivery of drug molecules to the brain parenchyma [39,40]. Various lipid-based drug delivery systems, such as solid lipid NPs, nanostructured lipid carriers, liposomes, niosomes, and cubosomes, have been explored for this purpose [38]. In addition to using materials that can enhance BBB penetration, the biodegradability of the material can play an important role in determining the rate of drug release from the NP and the control of the drug pharmacokinetics, thus mitigating unwanted drug accumulation and its potential side effects [41] and promoting better biocompatibility [42]. The three primary classes of biodegradable polymers include polyesters, polysaccharides (such as chitosan and cellulose), and poly(alkylcyanoacrylates) (such as poly(butyl cyanoacrylate) and poly(isohexyl cyanoacrylate)) [43,44,45,46].

### 2.3. Surface Charge

Another crucial parameter that modulates the passage of NPs across the BBB is surface charge. Surface charge can influence the peripheral tissue uptake of NPs circulating in the blood as well as the interaction of NPs with the BBB endothelium. Positively charged NPs have been found to show an increased cellular uptake rate compared to negatively charged and neutral NPs as examined across eight human cell lines [47]. BBB endothelial cells are more negatively charged because they harbour a higher quantity of proteoglycans, suggesting that positively charged NPs are more likely to be transferred through the BBB [48,49]. Using zeta potential measurements in bovine brain capillary endothelial cells through dynamic light scattering, it was shown that brain endothelial cells have a higher density of anionic charges, resulting in the ease of BBB penetration by lipophilic cationic compounds [48,49]. In addition, the final depth of tissue penetration can also vary depending on surface charge. For example, while positively charged gold NPs (+30 mV) may be preferable for drug delivery due to their higher level of uptake by proliferating cells, negatively charged NPs (−36 mV) may be more effective in delivering drugs deeper into the tissues because they have a higher diffusivity [50]. Furthermore, it is worth noting the toxicity and the potential compromise of BBB integrity that are associated with charged NPs [51]. In male Fischer-344 rats, it was shown that neutral NPs and low concentrations of anionic NPs do not compromise BBB integrity. On the other hand, high concentrations of anionic NPs and cationic NPs can disrupt the BBB within a relatively short timeframe [51]. It was postulated that the toxicity of anionic NPs is attributed to the negatively charged surfactant, while the toxicity of positively charged NPs is ascribed to accumulation of [14C]-sucrose in the BBB endothelium [51]. The effect of surface charge in determining the efficiency of NP-mediated brain targeting remains elusive and warrants further investigation.

### 2.4. Surface Modification

**Figure 3:**
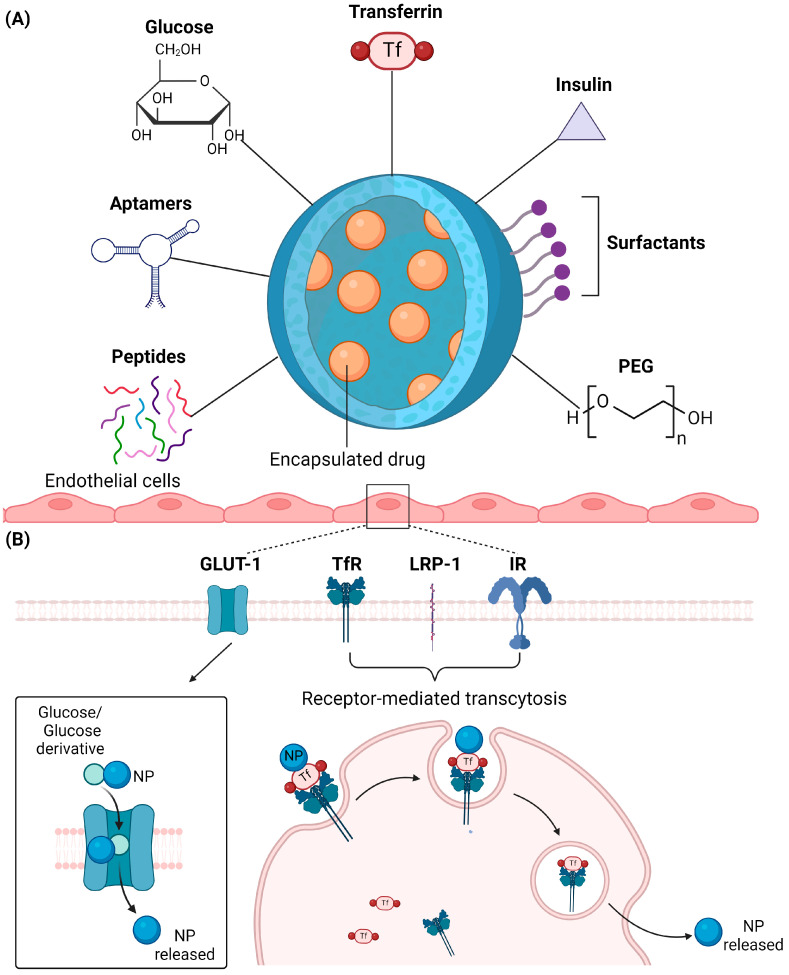
NP surface modification and mechanisms of NP transport through endothelial cells. (**A**) Ligands and chemical groups utilized for NPs include glucose, transferrin, insulin, aptamer, peptides, polyethylene glycol (PEG), and surfactants. These surface functionalization groups facilitate NPs’ entry through the blood–brain interface and uptake by the brain. (**B**) NPs with ligands and chemical groups can be recognized by cell surface receptors such as glucose transporter (GLUT-1), transferrin receptor (TfR), low-density lipoprotein receptor-related protein 1 (LRP-1) receptor, and insulin receptor (IR), which facilitate transcellular NP transport through different mechanisms. Schematics were created with BioRender.com.

Surface modification or conjugation of active functional groups to NPs represents an additional strategy for modulating efficiency in BBB penetration. This approach is particularly valuable when optimizing the inherent physicochemical properties of NPs alone proves insufficient to achieve the desired targeting efficiency. Furthermore, NPs have a high surface-to-volume ratio, which is ideal for functionalization with ligands or active functional groups to enhance BBB targeting [17,52]. Strategies to conjugate the NPs with biological or synthetic molecules, or to coat NPs’ surfaces with surfactants, have been developed to enhance the binding of NPs with specific BBB endothelial receptors or decrease the level of systemic clearance [53,54,55]. Importantly, it is feasible to finetune the administration of NPs to reach specific brain regions by targeting the higher expression levels of specific receptors in distinct neuronal populations [53,54]. It is also important to note that subtle deviations in density can have an impact on the drug delivery of conjugated NPs [56,57]. Below, we provide a summary of the properties and conjugation methods for various categories of ligands commonly used for the functional modification of NPs (Figure 3A).

#### 2.4.1. Glucose

Conjugation of NPs with glucose or related derivatives enhances BBB penetration by leveraging their binding affinity to specific glucose transporters on the endothelial cell surface. Specifically, glucose transporter type 1 (GLUT1) constitutes the subtype that is mainly expressed in the brain endothelium and principally responsible for glucose uptake [58]. Glucose-coated gold NPs have been shown to be transferred three times faster in cerebral endothelial cell lines compared to non-brain endothelial cell lines [59]. In the same study, a 3D co-culture system of primary human astrocytes and human endothelial cell lines was utilized, and it was shown that the glucose-conjugated NPs were accumulated within the astrocytes [59]. Another study provided evidence for a more effective internalization of 2-deoxy-D-glucose-coated NPs into an RG-2 glioma cell line compared to non-glycosylated counterparts [60]. These NPs were loaded with paclitaxel and displayed a higher intra-tumoral accumulation and a correspondingly higher therapeutic efficacy in orthotope glioma-bearing mice [60]. Glucose derivatives such as maltobionic acid can also be conjugated to telodendrimer-based NPs to enhance uptake through the BBB [61]. In mice bearing orthotopic patient-derived xenografts of diffuse intrinsic pontine glioma, NPs conjugated with maltobionic acid promoted BBB penetration through GLUT1-mediated transcytosis and increased the survival of the mice [61] (Figure 3B). It is worth noting that GLUT1 expression levels vary depending on different CNS pathological conditions. For example, it has been reported that GLUT1 is expressed at low levels in the cortex and hippocampus in human AD brains [62]. In glioblastoma, GLUT1 has been found increased within hypoxic necrotic areas, as opposed to the better oxygenated invasive borders and low-grade gliomas, where a decreased expression has been observed [63].

#### 2.4.2. Transferrin

Transferrin is an essential iron transporter abundant in the BBB endothelium, rendering it a good mediator for targeted drug delivery in the brain [64]. NPs conjugated with transferrin receptor (TfR)-targeting ligands or antibodies can promote their transcytosis across brain endothelial cells via receptor-mediated endocytosis. PEGylated albumin NPs with anchored transferrin on their surface have shown increased uptake and localization in the brains of healthy Wistar strain albino rats when intravenously administered [65]. Gold NPs with transferrin conjugation enhanced brain localization when systemically administered to BALB/c mice, and a larger amount of transferrin enabled strong attachment of gold NPs to brain endothelial cells [66]. However, although many NPs bound to the cells, they were mostly excluded from entering the brain. A follow-up study by the same group further optimized the NPs’ conjugation with an acid-cleavable linkage that allows the release of NPs from transferrin that is bound to the brain endothelial cells’ TfRs to facilitate their entry into the cells, allowing for increased delivery efficacy [67]. Transferrin-modified magnetic NPs have also been applied to promote the entry of small interference RNA against polo-like kinase I (siPLK1) into the brain in the context of glioblastoma [68]. The study showed an enhanced cellular uptake of siPLK1 in conjunction with an increased cytotoxic effect on the U87 glioblastoma cell line. Furthermore, the application of transferrin-modified magnetic NPs has demonstrated the accumulation of siPLK1 within the brain tissue of glioblastoma-bearing mice, together with a significant reduction in the tumor mass and improved survival of the mice [68]. Other transferrin-conjugated NPs have been reviewed elsewhere [69].

#### 2.4.3. Insulin

Another well-studied ligand important for BBB transport is insulin, which binds to insulin receptors (IR) on endothelial cells [70]. Ulbrich et al. conjugated insulin onto human serum albumin NPs and induced significant antinociceptive effects in mice [71]. In another instance, a monoclonal antibody that targets IR was modified on solid lipid NPs to improve the brain-targeting delivery of saquinavir [72]. Insulin-coated gold NPs (INS-GNPs) were synthesized to serve as a BBB transport system. INS-GNPs were found in mouse brains at five times greater concentrations than control untargeted GNPs. In addition, INS-GNPs can serve as computed tomography contrast agents to highlight specific brain regions in which they accumulate [73]. In a follow-up study, it was found that the 20 nm INS-GNPs had the highest accumulation within the brain, in line with the optimal size range discussed in earlier sections [53].

#### 2.4.4. Polyethylene Glycol (PEG)

The conjugation of NPs with PEG impacts the biodistribution of NPs by enhancing the efficacy of systemic delivery [74]. PEG is a hydrophilic molecule which provides a shield that prevents the interaction of circulating NPs with other blood components, thereby reducing opsonization and phagocytosis, which lead to their clearance from circulation [74]. Hence, PEGylation reduces uptake of NPs by the reticuloendothelial system and increases the probability of NPs reaching the BBB to interact with the brain endothelium. An added advantage of PEGylation is that it can act as a linker for ligand molecules to achieve active targeting to the brain [75]. These ligands include transferrin that binds to transferrin receptors on brain endothelial cells, lactoferrin that targets lactoferrin receptors, and other BBB-targeting peptides like arginyl-glycyl-aspartic acid (RGD), which target α_v_β_3_ integrin receptors [75]. Furthermore, PEGylation of NPs potentially decreases their neurotoxicity. In a study, lipid NPs were shown to induce neurovascular damage when injected into THY1-YFP transgenic mice via activation of caspase-1 and IL-1β [76]. PEGylation of lipid NPs abrogated P2X-caspase-1/IL-1β signaling in microglia, which reduced neuroinflammation and neurovascular damage [76].

#### 2.4.5. Peptides

Conjugating peptides with NPs enables them to bind to receptors and other proteins expressed on BBB endothelial cells, facilitating penetration of the BBB [77]. Typical examples of peptides targeting receptors that have been used in brain disorders include the RGD peptide targeting α_v_β_3_-integrin, which is highly expressed on tumor tissues [78]. In a study by Lou et al., cationic polymers constructed via RAFT copolymerization of *N*-(2-hydroxypropyl)-methacrylamide and *N*-acryloxysuccinimide conjugated with PEG-RGD peptides self-assembled with small interfering RNA (siRNA) to form NPs of 40 nm in size and displayed a two-fold increase in cellular uptake in glioblastoma cell lines [79]. Similarly, conjugation of a cyclic RGD ligand to poly(ethylene glycol)-block-poly(lactic acid) polymeric micelles enabled efficient transport across the intact BBB of normal mice and was able to also specifically target glioma cells of intracranial glioma-bearing nude mice [80]. Angiopep-2 (TFFYGGSRGKRNNFKTEEY) targets low-density lipoprotein receptor-related protein 1 (LRP-1) that is overexpressed by both endothelial cells of the BBB and glioma cells and has been used to coat hyaluronic acid NPs to enhance delivery to human glioblastoma cells [81]. While targeting LRP-1 on endothelial cells increases BBB transcytosis, studies have also shown that LRP-1 on the abluminal side of the BBB can clear LRP-1 targeting therapeutics [82]. To circumvent abluminal LRP-1-mediated clearance when utilizing angiopep-2 for brain targeting, a fusion peptide, K-s-A, was developed. This peptide responds to matrix metalloproteinase-1 cleavage on the abluminal side of the BBB, triggering the release of angiopep-2 to evade abluminal LRP-1-mediated clearance [82]. The peptide G23 has also been shown to promote BBB transcytosis upon binding to the ganglioside GM1 [83]. G23-coated curcumin-loaded zein NPs exhibited an increased transcytosis level as well as antitumor activity in a glioma cell line and in a 3D tumor spheroid model [84]. In a study using amyloid precursor protein/presenilin 1 (APP/PS1) transgenic mice, PEGylated NPs were loaded with siRNA and coated with the CGN peptide for BBB penetration and the Tet1 peptide for neuron-specific targeting to decrease the activity of the β-site amyloid precursor protein-cleaving enzyme 1 (BACE1) [85]. This construct led to a decrease in *BACE1* mRNA levels which was phenotypically translated into improved cognition in mice. Glutathione (GSH, L-γ-glutamyl-L-cysteinyl-glycine), a tripeptide, has also been used as an adjunct to facilitate drug delivery into the brain. GSH PEGylated liposomes were able to enhance BBB targeting efficacy and deliver higher amounts of amyloid beta-binding llama single-domain antibody fragments to a mouse model of AD [86]. Other types of peptides and conjugation strategies for NPs have been reviewed elsewhere [87].

#### 2.4.6. Aptamers

Aptamers constitute single-stranded oligonucleotides (DNA or RNA) that fold into defined 3D architectures and bind to specific molecules. They are typically obtained through combinatorial chemical technology, termed systemic evolution of ligands by exponential enrichment, against specific targets. In the past few years, aptamers have emerged as another conjugate for nanocarriers to promote their transport through the BBB, and recently a new human BBB shuttle aptamer, hBS, was discovered [88]. In a BBB microenvironment with human brain microvascular endothelial cells, astrocytes, and pericytes, the hBS aptamer was transported efficiently across the BBB through clathrin-mediated endocytosis [88]. Gold NPs conjugated to the aptamer U2 showed enhanced uptake in glioblastoma-bearing Balb/c nude mice compared to unconjugated gold NPs, thereby prolonging survival of the mice [89]. In another study, Gint4.T aptamer, which targets the platelet-derived growth factor receptor β, was conjugated to polymeric NPs containing a PI3K-mTOR inhibitor. In nude mice bearing intracranial U87MG tumor xenografts, there was increased accumulation of these polymeric NPs in mice brains compared to those without Gin4.T aptamer conjugation [90]. The existing evidence on aptamers and their role in NPs’ delivery stems from the field of glioblastoma treatment, but in the future the focus should also be moved to neurodegenerative disorders, especially after the recent advances in developing aptamers against amyloidogenic proteins [91].

#### 2.4.7. Surfactants

In addition to chemical modifications to the material backbone of NPs, surfactants that coat the surface of NPs can play a role in modulating BBB penetration. For example, PLGA NPs co-delivering methotrexate and paclitaxel were coated with the surfactants polyvinyl alcohol and poloxamer 188, which facilitated NP uptake by U-87 MG (human glioblastoma) and B65 (rat neuroblastoma) cell lines [92]. PEG surfactant has also been shown to increase internalization of PLGA NPs in the brains of Sprague–Dawley rats, where they were internalized within neurons and microglia [55]. In addition, NPs coated with the surfactants poloxamer 407 and polysorbate 80 have also demonstrated uptake in BBB endothelial cells and enhanced accumulation within the brain [55].

## 3. In Vitro and In Vivo Models of BBB and Ways to Detect BBB Penetration

### 3.1. In Vitro Transwell Models

While animal studies are advantageous since they comprise all the components and factors that modulate BBB penetration, they tend to suffer from large biological variability [93] and are typically tedious and expensive [94]. To circumvent these limitations, a broad variety of in vitro models have been developed over the last few decades with increasing complexity and anatomical accuracy. Comprehensive reviews have been published on these in recent years [95,96,97], and the key models are summarized here.

**Figure 4:**
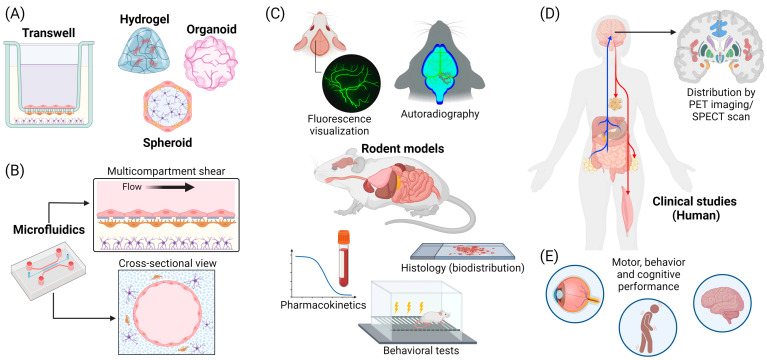
Preclinical and clinical models of the BBB for NP penetration studies. (**A**) Transwell-based BBB models are widely used, with more complex models achievable using hydrogels, spheroids, or organoids. (**B**) Microfluidics allows for the introduction of shear flow while maintaining compartmentalization of BBB cells. Novel fabrication methods enable the production of anatomically accurate vessels with circular cross-sections. (**C**) Animal studies typically employ rodent models. NP uptake can be directly observed via fluorescence visualization or autoradiography or indirectly assessed through pharmacokinetic assays, histology-based biodistribution studies, or behavioral tests. (**D**) Clinical studies to assess NP BBB penetration are typically conducted using positron emission tomography (PET) imaging or single-photon emission computed tomography (SPECT) scans of human brains. Arrows indicate direction of blood flow in (blue) and out (red) of the brain. (**E**) Motor, behavioral, and cognitive tests are conducted on human subjects to determine the efficiency and effectiveness of therapeutic targeting. Schematics were created with BioRender.com.

The simplest in vitro BBB model involves the use of the two compartment transwell setup, which is comparably straightforward to assemble (Figure 4A) [98]. Primarily, BBB endothelial cells are grown as a monolayer on the porous membrane insert, while other astrocytes, pericytes, and other neural cells are grown in the basolateral compartment [99,100,101]. While this arrangement lacks direct cell-to-cell communication between pericytes, astrocytes, and endothelial cells, it promotes BBB regulation indirectly via secreted soluble proteins. However, this model is limited by the fact that NPs can adhere to the membrane filter and become trapped within its pores, thus preventing effective analysis of the transendothelial delivery of NPs [102]. To overcome the issue with this model, a filter-free transwell model has been developed which consists of a collagen gel covered with a monolayer of brain microvascular endothelial cells. The transendothelial delivery of PEG-P(CL-*g*-TMC) polymersomes that were functionalized with GM1-targeting peptides was assessed by fluorescence microscopy, and it was confirmed that this system allowed for more NP transcytosis compared to that of a conventional transwell filter system [103].

A monolayer of cells fails to replicate the intricate organization seen at the BBB, lacking the complex architecture inherent in its structure. In contrast, 3D cell cultures can accurately mimic the neurovascular unit cells as well as the vasculature [104,105]. These structures are typically generated by co-culturing cells from the neurovascular unit within hydrogels that emulate the extracellular matrix, through self-assembly in culture media, or by employing precision-engineered microstructures designed to simulate the 3D physiological architecture (Figure 4A) [97]. In a recent example, Singh et al. developed a hydrogel-based BBB using a collagen hydrogel containing astrocytes overlaid with a monolayer of endothelium cells on a transwell setup. The endothelial monolayer had transendothelial electrical resistance values and expressed tight-junction markers typical of the physiological BBB. This model was used to monitor transport of glycoproteins [106]. Vasculogenesis is also possible within the hydrogel through invasion of tip cells from the BBB endothelial monolayer or self-asembly of the endothelial cells seeded in the hydrogel [107]. The use of hydrogels provides an extracellular matrix (ECM) where cells can establish cell–cell and cell–ECM interactions in a 3D environment [108]. These hydrogel models can be formed on standard culture plates, obviating the use of specialized equipment, which makes them an affordable option for the development of 3D BBB models. However, hydrogel-based models suffer from disadvantages such as difficulty in optimizing the mechanical properties of the hydrogel for suitable cell growth and limited contact between the various cell types.

Spheroids or organoids are formed by a collection of organ-specific cell types capable of self-assembly and self-organization akin to in vivo conditions and have been used as models to study BBB penetration (Figure 4A) [109,110,111,112]. Unlike hydrogel-based models, spheroids lack supporting structures or scaffolds, maximizing direct cell–cell contact among neurovascular unit cells. To fabricate spheroid-based models, a suspension of neurovascular unit cells is seeded into low-attachment or hanging-drop culture plates to allow cells to self-assemble [109]. In addition, human BBB organoids can be produced at a high throughput using micro-patterned hydrogels. This technique enables the generation of BBB organoid arrays, facilitating the simultaneous growth of over 3000 uniform organoids per experiment with good reproducibility [110]. Generally, endothelial cells, astrocytes, and pericytes are the most traditional neurovascular unit cells used in the construction of spheroids. However, alternative cells such as neurons, microglia, or oligodendrocytes can be included to increase the grade of physiological relevance. For example, spheroids comprising astrocytes, microglia cells, oligodendrocytes, and neurons with a surface layer consisting of pericytes and endothelial cells were used to study the penetration of gold NPs [111]. In another instance, BBB spheroids comprising microvascular endothelial cells, brain vascular pericytes, and astrocytes combined with primary cortical neurons and microglia isolated from neonate rats were used to study the transport of CS-PMMA30:PVA-PMMA17 NPs across them. While most of the NPs accumulated on the spheroid surface, some NPs permeated into the spheroids and suggested the possible involvement of astroglia or microglia in the transport of CS-PMMA30:PVA-PMMA17 NPs [112].

### 3.2. In Vitro Microfluidic Models

The transwell model is generally unable to fully reproduce the complexity of the BBB, including aspects such as shear flow and vascular geometry, resulting in only a modest estimation of in vivo BBB permeability [113]. Physiological levels of fluid shear have been shown to have a protective effect on the BBB through reinforcement of the tight junctions and suppression of inflammation, while elevated shear has been linked to BBB disruption through tight junction degradation [114]. Microfluidic models of the BBB have been developed to allow for fluid flow to be supplied (Figure 4B). These models typically comprise a main flow channel lined with cultured BBB endothelial cells flanked by a secondary channel containing the supporting cell types, interfaced by a porous polymeric membrane [115,116]. BBB microfluidic chips with shear flow minimize nonspecific NP binding and accumulation on the side walls and allow for the direct visualization of NP localization, which offers the possibility of studying NP transport and trafficking [117,118]. Examples of NPs studied using such models include high-density lipoprotein-mimetic NPs with apolipoprotein A1 and gH625 peptide-conjugated polystyrene NPs [117,118].

Due to the nature of the microfluidic device fabrication procedure, their configurations are usually sandwich-like and planar and thus lack the curvature representative of blood vessels as described above. To this end, upgraded 3D configurations of the microfluidic BBB models have been developed to produce cylindrical flow channels which are more biomimetic. For example, a microfluidic model with a cylindrical channel generated by ‘‘viscous fingering” was manufactured, which entails the prefilling of the microchannel with hydrogel followed by displacement of the circular channel by a hydrostatically driven flow of culture media [119]. In other instances, soluble microneedles were used to construct an array of parallel microchannels within a collagen hydrogel [120], and ultra high-resolution two-photon lithography was used to reproduce the capillary network in the BBB anatomy [121].

### 3.3. In Vivo Models

While the in vitro models discussed in preceding sections provide the advantages of reproducibility and straightforward experimental comparisons, they fall short of the physiological intricacy achievable only through in vivo models. Animal models offer a platform for effectively exploring BBB penetrance and physiological regulatory mechanisms. Studies on NP penetration have predominantly utilized rodent models, including mice (e.g., Balb/c, Kunming, ICR, FVB/Ntac) and rats (e.g., P18, Sprague–Dawley, Wistar, Fischer-344) (Figure 4C) [122]. Access points for NP infusion include the carotid artery, femoral vein, tail vein, and jugular vein, as well as direct injection into the heart. NP penetration is then typically assessed qualitatively via direct fluorescence visualization or behavioral tests, or quantitatively using autoradiography, pharmacokinetic blood plasma measurements, microdialysis, or histological biodistribution studies [122]. For instance, a rodent model was employed to examine the BBB penetrance of carbamazepine-loaded methoxy poly(lactide-co-glycolide)-b-poly(ethylene glycol) methyl ether NPs [123]. The efficacy of focused ultrasound in enhancing NP delivery into the brain was explored in a mouse model [124]. In this study, 8 week-old ICR mice received injections of gold NPs and were sacrificed post-ultrasound exposure. Subsequently, their brains were collected, digested in nitric acid, and analyzed for NP uptake using inductively coupled plasma–optical emission spectrometry [124]. APP/PS1 double transgenic C57BL/6 mice were used to evaluate the efficacy of Prussian blue/polyamidoamine dendrimer/angiopep-2 NPs in crossing the BBB and regulating microglia function for the treatment of AD [125]. A middle cerebral artery occlusion ischemic stroke mouse model was also used to study the delivery of microglia-targeting lipid NPs to the brain [126]. Histological and neurobehavioral tests can also be used to assess NP efficacy, as shown in the delivery of dopamine-loaded NPs across the BBB of 6-hydroxydopamine PD mice [127].

### 3.4. Clinical Detection of BBB Penetration

In a clinical setting, BBB penetrance and the entry of therapeutics into the BBB parenchyma can be evaluated based on clinical, electrophysiological, and imaging techniques. These methods include positron emission tomography (PET), single-photon emission computerized tomography (SPECT), and cerebrospinal fluid (CSF) sampling to indirectly measure BBB penetration (Figure 4D) [128]. PET enables the visualization of the localization of radiolabelled drugs in the brain, and an improved technique measures displacement of a validated PET tracer from a receptor, which can provide direct evidence of both BBB penetration and binding to the relevant receptor. SPECT is a less expensive alternative to PET, but it is also more difficult to quantify reliably enough for predictions of drug concentrations in the CNS. Future studies could potentially integrate these tracers in NP-mediated drug delivery to monitor BBB penetration. For CSF sampling, it is generally accepted that if a compound reaches the CSF, it has the propensity to reach the brain too. Unfortunately, obtaining a CSF sample by lumbar puncture or spinal catheterization can be distressing, causing post-puncture headache in 5–50% of cases [129], and does not definitively demonstrate that pharmacologically active levels of the compound reach the target tissue. Recently, a battery of drug-sensitive CNS tests called NeuroCart has been developed to determine the efficiency and effectiveness of drug targeting (Figure 4E) [128]. NeuroCart entails pharmacodynamics tests that capture CNS function across six domains including executive function, attention, memory, visuomotor function, motor skills, and subjective drug effects, and has been shown to display a clear concentration-dependent relationship with the administered drug [128]. Although not yet fully established, NeuroCart represents a useful tool to confirm if a drug (NPs that are either non-conjugated or conjugated) can penetrate the BBB. The list of in vitro and in vivo models of the BBB is summarized in Table 1.

## 4. Route of NP Administration and Uptake under Normal BBB Conditions

The most common routes of administering NPs to the brain include oral, transdermal, intravenous (systemic), and inhalation methods [130,131], each with its own set of advantages and disadvantages. Oral and transdermal administration offer non-invasive NP delivery options. Generally, the oral route is preferred due to its convenience, painlessness, and lower risk of infection and needle injuries [132]. Inhalation and transdermal deliveries offer additional benefits such as the large surface area of the skin and lung endothelium, as well as localized action without the risk of systemic side effects [130]. In comparison, the intravenous route (systemic administration) offers the major advantage of rapid onset of action and bypasses first-pass metabolism or degradation by proteolytic enzymes in the gastrointestinal tract [131]. First-pass metabolism in the liver leads to decreased drug concentrations in the blood and subsequently at the BBB, representing the primary disadvantage of oral delivery, along with reduced absorption due to a damaged gastrointestinal epithelium [130,133]. However, the intravenous route may be considered more invasive compared to others, as it requires venipuncture. Intranasal administration has garnered increased interest recently due to its potential for direct transport into the brain via olfactory neurons, bypassing the BBB restriction [134]. This method is less invasive compared to others, although it is hindered by ciliary movements, which can dampen absorption despite the dense network of blood vessels in the nasal mucosa [135].

In addition to various routes of administration, emerging technologies facilitate the uptake of NPs across the BBB locally. Focused ultrasound (FUS), generated from a curved transducer or phased array, has become a common method for enhancing drug delivery by transiently opening the BBB [136]. FUS-induced BBB opening allows NP-based drug delivery systems to efficiently reach the brain. Furthermore, photoacoustic brain imaging using NP-based contrast agents effectively visualizes brain morphologies or diseases. The application of FUS-mediated NP delivery has been extensively reviewed [136]. In addition to leveraging technological adjuncts, NP delivery can be enhanced through co-delivery or combination strategies. For example, a co-delivery system employing methotrexate and paclitaxel loaded simultaneously into PLGA NPs facilitated their uptake into brain cell lines [92]. Upon reaching the BBB, NPs can enter the brain parenchyma through either passive or active transport mechanisms. Small lipid-soluble particles may passively diffuse through endothelial cell membranes or via paracellular transport across tight junctions [17,137]. However, passive diffusion is less common, as the majority of NPs are taken up through carrier-mediated, receptor-mediated, or adsorption-mediated transport mechanisms [17,137]. To facilitate carrier- and receptor-mediated transport, NPs themselves or conjugated molecules on their surface bind with high affinity to carriers or receptors. In carrier-mediated transport, transport proteins anchored in the cell membrane facilitate the entry of NPs across the plasma membrane into the cytoplasm. In receptor-mediated transport, NPs binding to specific receptors on the cell membrane cause membrane invagination and the formation of endosomes containing the NP-receptor complex. Adsorption-mediated transport operates similarly, often via clathrin-mediated endocytosis, with intracellular vesicles transported to the basal membrane, although initial interactions are mediated by electrostatic forces between positively charged NPs and negatively charged cell membranes [17,137]. To overcome challenges associated with BBB-specific delivery, intraparenchymal and intraventricular delivery strategies can be used, which have the advantage of direct (local) delivery into the brain [138]. However, these procedures necessitate invasive neurosurgery, limiting their applicability [138]. Therefore, formulating an optimal NP delivery vehicle requires careful consideration of the route of administration, physicochemical characteristics, and functionalization affecting cellular uptake routes.

## 5. Effect of BBB Breakdown on NP-Mediated Drug Delivery

**Figure 5:**
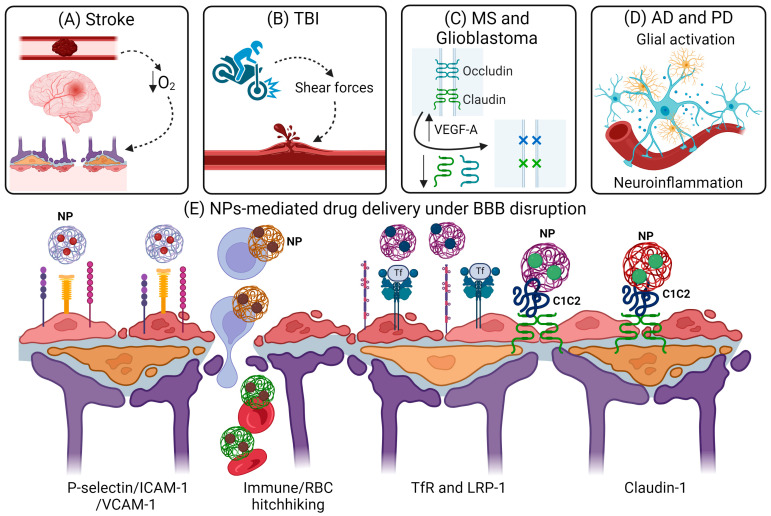
BBB disruption and NP-mediated drug delivery for the treatment of neurological diseases. (**A**–**D**) Mechanisms of BBB disruption in (**A**) stroke due to increased oxidative stress, (**B**) TBI due to shear force-induced mechanical injury, (**C**) MS and glioblastoma due to upregulation of VEGF-A and downregulation of tight junction proteins, and (**D**) AD and PD due to inflammatory cytokine release and neuroinflammation. (**E**) Cell junction or tight junction proteins, as well as membrane receptors, that are overexpressed on the BBB endothelium promote the uptake of NPs into the brain. Immune cell and red blood cells (RBC) hitchhiking represent new strategies for coating NPs onto immune cell or RBC membranes to facilitate NP delivery across the BBB. Schematics were created with BioRender.com.

Under normal conditions, the BBB serves as a robust shield, safeguarding the CNS from potentially neurotoxic substances. However, in pathological conditions such as stroke, TBI, and neurodegenerative disorders, the BBB becomes compromised, losing its protective function as a physical barrier (Figure 5A–D). In ischemic stroke, elevated oxidative stress, involving reactive oxygen species, damages the BBB (Figure 5A) [139]. TBI induces acute damage to blood vessels due to shear forces, resulting in BBB disruption (Figure 5B) [140]. In MS, decreased expression of tight junction proteins and adherens junction proteins indicates BBB breakdown, coupled with increased expression of endothelial adhesion molecules facilitating infiltration of immune cells into the CNS (Figure 5C) [141]. Glioblastoma cells secrete growth factors like vascular endothelial growth factor (VEGF), promoting abnormal angiogenesis and dysfunctional blood vessels, thereby damaging the BBB (Figure 5C) [142]. AD is associated with BBB dysfunction, attributed to protein aggregate deposition, leading to heightened inflammatory responses and the degeneration of smooth muscle cells, pericytes, and endothelial cells (Figure 5D) [143]. In PD, PET scans and post-mortem analyses reveal increased BBB permeability alongside elevated levels of pro-inflammatory cytokines (Figure 5D) [144]. Overall, BBB disruption triggers ion dysregulation, edema, and neuroinflammation, culminating in neuronal dysfunction, heightened intracranial pressure, and neuronal degeneration [145].

Although a dysfunctional BBB is typically undesirable, researchers have leveraged its properties for nanoparticle-mediated drug delivery. The primary mechanism facilitating the transport of NPs across an impaired BBB involves targeting molecules on the brain endothelium that are highly expressed under pathological conditions. An inflamed endothelium, characteristic of neuroinflammation and glioblastoma, often overexpresses molecules such as P-selectin, intercellular adhesion molecule 1, and vascular cell adhesion molecule 1, promoting leukocyte adhesion (Figure 5E) [146,147]. This heightened expression increases the likelihood of targeted NPs accumulating within the brain [146,147]. In addition, transferrin receptors and LRP-1 are upregulated in dysfunctional the BBB in glioblastoma, and conjugated NPs targeting these receptors have been developed for enhanced drug delivery in glioblastoma cell lines and orthotopic mouse models [148,149]. In a stroke model, research demonstrated upregulation of claudin-1 in the BBB, indicative of an inflammatory phenotype in the brain endothelium [150]. This overexpression of claudin-1 presents an opportunity for NP delivery targeting, as evidenced by another study showing C1C2-NP accumulation in brain endothelial cells with high levels of claudin-1 expression during aging [151]. Another method for NP delivery though the BBB lies in the loading of NPs on immune cells and red blood cells, which infiltrate the brain to a greater extent in pathological conditions, an approach that is termed immune “hitchhiking” (Figure 5E).

For instance, doxorubicin-loaded PLGA NPs conjugated to macrophages were systemically administered in mice with glioblastoma, resulting in substantial intracerebral accumulation and improved survival outcomes [152]. In a rat model of ischemia/reperfusion injury, cyclic RGD-liposomes, which bind to integrin receptors, were delivered using neutrophils and monocytes, leading to a reduction in the volume of the infarcted brain [153]. Interestingly, red blood cell membranes can also be utilized to coat NPs, enhancing BBB transcytosis. Red blood cell membrane-coated PLGA NPs bound to the D-peptide fragment of candotoxin, with a high affinity to nicotinic acetylcholine receptors, exhibited high accumulation in the brain in a glioma mouse model [154]. While it has been assumed that a compromised BBB facilitates NP entry via diffusion, brain regions affected by diseases may undergo pathological BBB breakdown, characterized by functional and structural alterations in blood vessels. These changes can hinder the effective delivery of therapeutic agents to the brain. Enlarged perivascular spaces accumulate blood-derived products, water, and electrolytes, disrupting the normal diffusion of drug molecules across brain extracellular spaces. This interference affects interstitial fluid formation and flow, impairing distribution throughout the CNS [155]. Therefore, restoring BBB function remains crucial, and leveraging BBB dysfunction for therapeutic delivery requires strategic consideration.

## 6. Summary and Future Perspectives

The study of NPs and their potential therapeutic applications in neurological disorders has undergone significant growth in recent years. This surge in interest stems from accumulating evidence that has enhanced our understanding of the physiological properties of the BBB and its alterations under pathological conditions. Leveraging these insights, there is a growing emphasis on utilizing these changes to facilitate more efficient delivery of drug-loaded NPs, thereby influencing disease progression. Hence, deepening our knowledge of NP characteristics that facilitate BBB targeting and entry into the brain is imperative. Here, we summarize the key properties of NPs, encompassing aspects such as size and shape, chemical composition, surface charge, surfactants, and conjugation with targeting ligands or molecules, all of which can be optimized to enhance BBB penetration. We also discuss various in vitro and animal models of the BBB that facilitate the development of NP delivery systems and enable the clinical detection of brain-targeting drugs. Under physiological conditions, the BBB acts as an important barrier that protects the brain from potentially harmful substances. However, in pathological states, BBB dysfunction may induce pathological changes contributing to disease onset while paradoxically offering opportunities to facilitate drug delivery alongside immune cell and blood product influxes.

Apart from delivering drugs to the brain, there is increasing research on using NPs as carriers for CRISPR-Cas9 and RNA delivery in the brain for genome editing and therapeutic purposes [156,157,158,159]. CRISPR-Gold, a gold nanoparticle, targeted the metabotropic glutamate receptor 5 (mGluR5) in a mouse striatum to reduce local mGluR5 levels after an intracranial injection, consequently reducing behavioural deficits [160]. In another instance, bio-reducible lipid NPs were used to deliver CRISPR components (Cre recombinase or anionic Cas9:single-guide (sg)RNA complexes) into mouse brain tissues for genome editing [161]. Using a glutathione-sensitive polymer shell incorporating a dual-action ligand that facilitates BBB penetration, CRISPR components have been efficiently encapsulated and delivered for gene therapy in glioblastoma mouse models [162]. Lipid NPs have also been utilized to deliver RNA therapeutics in microglial cells and to reduce neuroinflammation in mouse models [163].

While NPs have mainly been studied as carriers of drugs into the brain, NPs’ value extends beyond their function as delivery systems, as some may exhibit therapeutic properties themselves independent of drug loading or conjugation [20,26,27]. Examples of these NPs include PLGA NPs, acidic NPs, and metal-based NPs like zinc oxide and platinum NPs [20,26,27,164], and they have been applied in neurodegenerative diseases [165,166], although further optimization is needed to enhance their BBB penetration. Moreover, NPs, with their versatile properties, hold potential for personalized medicine. By tailoring NP properties to individual patient pathologies, specific and effective brain targeting can be achieved under pathological conditions, and their therapeutic efficacy can also be validated through omics technologies, representing a significant advancement in personalized therapeutic strategies for neurological disorders [167,168].

To conclude, the efficacy of drug delivery in neurological disorders is compromised by the BBB, which acts as a limiting factor despite its general protective functions. Hence, refining drug entry into the brain by overcoming the BBB acquires paramount importance and represents a challenge in modern pharmaceutics. That being said, NPs hold promise for the future and can play a vital role in circumventing this natural barrier. Current knowledge about the use of NPs for drug delivery is derived from cellular and animal models, but evidence from clinical studies is still lacking. NPs have begun to be used in clinical practice, mainly in cancer treatment, although their application in neurological disorders is gaining traction. The last decade has seen an increasing presence of brain-targeted NPs in clinical trials, which have been evaluated for their safety and efficacy either alone or in combination with existing clinical protocols (Table 2). These NPs have primarily served as drug carriers for direct therapeutic intervention or as imaging contrast agents for disease diagnostics and etiology. To promote the assimilation of NPs in clinical workstreams, it is necessary to understand their material properties and safety profiles, good manufacturing practices to ensure homogeneous size distributions and properties, and large-scale manufacturing for cost reduction. Overall, the use of NPs represents a highly promising therapeutic strategy for fine-tuned BBB targeting and drug delivery in neurological disorders.

## Figures and Tables

**Table 1:** In vitro and in vivo BBB models for investigation of NP penetration.

BBB Model	Features	Advantages	Limitations
In vitro BBB models			
Transwell	Two-compartment model comprising BBB endothelial cells grown as a monolayer on the porous membrane insert, while other astrocytes, pericytes, and other neural cells are grown in the basolateral compartment.	Simple to set up and allows for co-culturing of different cells in the neurovascular unit.	Monolayer of cells fails to replicate the organization and architecture of the BBB. NPs can be trapped in the membrane filter. Absence of fluid shear.
Hydrogel	Generated by co-culturing cells from the neurovascular unit within hydrogels that emulate the extracellular matrix, through self-assembly in culture media, or by employing precision-engineered microstructures designed to simulate the 3D physiological architecture.	The extracellular matrix enables cell–cell and cell–ECM interactions in a 3D environment, with possible vasculogenesis. Affordable procedure with minimal need for specialized equipment.	Difficulty in optimizing the mechanical properties of the hydrogel for suitable cell growth. Limited contact between various cell types. Absence of fluid shear.
Spheroid/organoid	Formed by a collection of organ-specific cell types capable of self-assembly and self-organization akin to in vivo conditions.	Direct cell–cell contact among neurovascular unit cells. Ease of multiplexing using spheroid/organoid arrays.	No current demonstration of direct quantification of NP transport. Absence of fluid shear.
Microfluidic	Typically comprises a main flow channel lined with cultured BBB endothelial cells flanked by a channel containing the supporting cell types, interfaced by a porous polymeric membrane. 3D upgrades are available with biomimetic cylindrical blood vessels.	Inclusion of fluid shear to reproduce physiological conditions. Direct visualization of NP localization is possible.	Microfabrication process is tedious and resource-intensive. Sophisticated skillset required for microchannel assembly and cell culture.
In vivo BBB models			
Mice (Balb/c, Kunming, ICR, FVB/Ntac)	NPs are administered and assessed qualitatively via direct fluorescence visualization or behavioral tests or quantitatively using autoradiography, pharmacokinetic blood plasma measurements, microdialysis, or histological biodistribution studies	Reproduces the true physiological complexity in terms of architecture and cellular composition. Available disease models such as AD, PD, and stroke.	Large biological variability between animals reduces reproducibility of findings. Tedious and expensive to maintain animals, requiring stringent study planning and animal welfare considerations.
Rat (P18, Sprague Dawley, Wistar, Fischer-344)			
Human studies			
Clinical trials	NP penetrance and entry into the BBB parenchyma can be evaluated based on clinical, electrophysiological, and imaging techniques such as PET, SPECT, and CSF sampling.	Circumvents model fidelity limitations. Direct visualization of localization of radiolabelled drugs in the brain possible.	Radiological imaging techniques are expensive and have limited accuracy in quantifying drug concentrations in the CNS. Lumbar puncture for CSF sampling can be distressing and cannot conclude whether a compound has reached its target.

**Table 2:** Brain-targeted NPs in various phases of clinical trials.

NP Used	Identifier (Start Year)	Study Scope	Phase/Status
Curcumin NP	NCT02104752 (2014)	Test whether curcumin NPs will improve behavioral measures and biomarkers of cognition and neuroplasticity in patients with schizophrenia who have been prescribed antipsychotics.	1,2/Completed
Ultrasmall superparamagnetic iron oxide NP	NCT02549898 (2015)	Investigate inflammation of cranial and meningeal arteries during pharmacologically induced migraine attacks using black blood imaging MRI.	-/Completed
Ultrasmall superparamagnetic iron oxide NP	NCT02511028 (2015)	Investigate the safety of ferumoxytol and examine the spatial and temporal enhancement patterns of ferumoxytol compared to patterns seen with gradient-echo imaging and gadolinium contrast in multiple sclerosis lesions.	1/Completed
NU-0129 spherical nucleic acid gold NP	NCT03020017 (2017)	Assess the safety of intravenous NU-0129 in patients with recurrent glioblastoma multiforme or gliosarcoma.	1/Completed
MTX110 NP	NCT03566199 (2018)	Study the side effects of panobinostat nanoparticle formulation MTX110 in treating participants with newly-diagnosed diffuse intrinsic pontine glioma.	1,2/Completed
CNM-Au8 gold nanocrystals	NCT03993171 (2019)	Assess the CNS metabolic effects, safety, pharmacokinetics, and pharmacodynamics of CNM-Au8 in patients who have been diagnosed with multiple sclerosis.	2/Recruiting
CNM-Au8 gold nanocrystals	NCT04098406 (2019)	Assess the efficacy, safety, and pharmacokinetics/pharmacodynamics effects of CNM-Au8 as a disease-modifying agent for the treatment of amyotrophic lateral sclerosis.	2/Completed
CNM-Au8 gold nanocrystals	NCT03815916 (2019)	Assess the CNS metabolic effects, safety, pharmacokinetics, and pharmacodynamics of CNM-Au8 in patients with PD.	2/Completed
MTX110 NP	NCT04264143 (2020)	Find the maximum tolerated dose of MTX110 (a water-soluble panobinostat nanoparticle formulation) and gadolinium that can be given safely in children with newly diagnosed diffuse midline gliomas.	1/Completed
NTLA-2001 lipid NP	NCT04601051 (2020)	Evaluate the safety, tolerability, pharmacokinetics, and pharmacodynamics of NTLA-2001 in participants with hereditary transthyretin amyloidosis.	1/Active, not recruiting
AGuIX Gd-based NP	NCT04899908 (2021)	Determine whether AGuIX Gd-based nanoparticles make radiation work more effectively in the treatment of patients with brain metastases.	2/Recruiting
AGuIX Gd-based NP	NCT04881032 (2022)	Evaluate the association of AGuIX nanoparticles with radiotherapy plus concomitant temozolomide in the treatment of newly diagnosed glioblastoma.	1,2/Recruiting
Ferumoxytol superparamagnetic iron oxide	NCT06146751 (2023)	Evaluate the effectiveness of a novel ferumoxytol-enhanced cardiac magnetic resonance in detecting intracardiac thrombus in patients with ventricular aneurysm and after percutaneous ventricular reconstruction.	-/Recruiting
NanoTherm ASI iron oxide NP	NCT06271421 (2024)	Evaluate the efficacy and tolerance of using the NanoTherm therapy system in recurrent glioblastoma multiforme	-/Recruiting

## Data Availability

Not applicable.
